# Blocking the IL-4/IL-13 Axis versus the JAK/STAT Pathway in Atopic Dermatitis: How Can We Choose?

**DOI:** 10.3390/jpm14070775

**Published:** 2024-07-22

**Authors:** Laura Calabrese, Martina D’Onghia, Laura Lazzeri, Giovanni Rubegni, Elisa Cinotti

**Affiliations:** 1 Dermatology Unit, Department of Medical, Surgical and Neurological Sciences, University of Siena, 53100 Siena, Italy; 2 Institute of Dermatology, Catholic University of the Sacred Heart, 00168 Rome, Italy

**Keywords:** atopic dermatitis, personalized medicine, JAK inhibitors, anti IL-4/13 monoclonal antibodies

## Abstract

Atopic dermatitis (AD) is an immune-mediated skin disorder with a chronic-relapsing course and a multifactorial pathogenesis. In contrast to the traditional concept of AD as solely a type 2 immune-activated disease, new findings highlight the disease as highly heterogeneous, as it can be classified into variable phenotypes based on clinical/epidemiological or molecular parameters. For many years, the only therapeutic option for moderate–severe AD was traditional immunosuppressive drugs. Recently, the area of systemic therapy of AD has significantly flourished, and many new substances are now marketed, licensed, or in the last step of clinical development. Biological agents and small molecules have enriched the therapeutic armamentarium of moderate-to-severe AD, such as dupilumab, tralokinumab, lebrikizumab (monoclonal antibodies targeting the IL-4/13 pathway), abrocitinib, upadacitinib, and baricitinib (JAK inhibitors). Indeed, the AD treatment paradigm is now split into two main approaches: targeting the IL-4/13 axis or the JAK/STAT pathway. Both approaches are valid and have strong evidence of preclinical and clinical efficacy. Therefore, the choice between the two can often be difficult and represents a major challenge for dermatologists. Indeed, several important factors must be taken into account, such as the heterogeneity of AD and its classification in phenotypes, patients’ comorbidities, age, and personal preferences. The aim of our review is to provide an overview of the clinical and molecular heterogeneities of AD and to explore the factors and parameters that, in clinical practice, may help inform clinical decision-making.

## 1. Introduction

Atopic dermatitis (AD) is one of the most common immune-mediated skin disorders, with a chronic-relapsing course and a multifactorial pathogenesis [1]. Prevalence of the disease is very high, affecting approximately 15–20% of children and 1–3% of adults worldwide. Furthermore, it varies widely across different countries and ethnic groups [2].

The disease is clinically characterized by itchy eczematous lesions primarily involving flexural areas, face, neck, and distal extremities, and it might precede other non-cutaneous atopic manifestations, such as asthma and allergic rhinitis (AR), priming the so-called “atopic march” [3]. Recent understanding of AD pathogenesis reveals that the disease is not solely driven by type 2 immune responses; rather, it is highly heterogeneous. Indeed, it can be subdivided into various phenotypes based on clinical, epidemiological, or molecular parameters [4].

For decades, the only therapeutic option for moderate-to-severe AD was traditional immunosuppressive agents such as cyclosporine. Nowadays, the field of systemic therapy for AD includes several new substances available, approved, or in the final stages of clinical development. Biological agents and small molecules have expanded the therapeutic arsenal for moderate-to-severe AD, including dupilumab (a monoclonal antibody targeting IL-4Rα), tralokinumab and lebrikizumab (anti-IL-13 monoclonal antibodies), abrocitinib and upadacitinib (JAK1 inhibitors), and baricitinib (a JAK1/2 inhibitor) [5]. Indeed, the AD treatment paradigm is now divided into two main approaches: targeting the T helper (Th) 2 pathway, especially the IL-4/13 axis, and targeting the JAK/STAT pathway. 

Both strategies are highly valid and have strong evidence of preclinical and clinical efficacy. Therefore, the choice between the two can often be difficult and represents a major challenge for dermatologists. In this choice, several important factors must be considered, such as the heterogeneity of AD and its classification in phenotypes, patients’ comorbidities, age, and personal preferences.

The aim of our review is to provide an overview of the clinical and molecular heterogeneity of AD and to explore the factors and parameters that, in clinical practice, may supportively inform clinical decision-making.

## 2. Heterogeneity of AD

AD is nowadays considered a highly heterogeneous inflammatory skin disorder. Skin lesions may considerably differ depending on several factors, such as body location or age, ethnicity, and stage of the disease [5]. Moreover, AD is not only phenotypically diverse, but is also characterized by a highly varied repertoire of endotypes, dictated by the predominant immune mechanism driving the pathogenesis. Additionally, the variable clinical course and degree of responsiveness of patients to therapies further endorse the heterogeneity in the mechanisms underlying AD [6].

For these reasons, much attention has recently been paid to the identification of different AD clinical phenotypes, as well as of molecular endotypes, that could lay the foundation for precise subtype-specific therapeutic approaches.

## 3. AD Phenotypes

The main phenotypes of AD are based on age-related clinical pictures, levels of IgE, disease stage, and body localization [7]. Furthermore, important phenotypical and immunological differences in AD have been described across various ethnic groups [8].

### 3.1. Age-Related Phenotypes

Typically, at least four different clinical pictures of AD have been defined according to the age of patients: infantile, childhood, adolescent/adult, and elderly [9]. 

In infants aged <1 year, AD is typically restricted to the face, where it is characterized by erythematous and highly pruritic lesions alongside moist, oozing papulo-vesicles that sometime form crusts or scales and can be concentrated in the perioral region, on the cheeks, or, in some cases, the scalp. A notable sparing of the diaper area is observed in infants [10]. In children, AD becomes more localized and commonly affects flexor surfaces with predominant oozing and crusting [11]. Adults may have only chronic hand AD or the head–neck–dermatitis type, which involves the upper trunk, shoulders, and scalp, presenting both acute exudative and lichenified lesions [11]. Skin manifestations in elderly AD are similar to those of adult AD, but the reverse sign of lichenified eczema (“reverse lichenification”) around unaffected folds of the elbows and knees is more common than the classic sign of localized lichenification in adults [12]. Interestingly, scientific evidence has highlighted that these changes might reflect background modifications of immune microenvironment over time. Indeed, several studies have demonstrated a strong Th2 skewing in pediatric AD [13,14,15,16]. A recent cross-sectional study evaluated age-specific changes in lesional and non-lesional tissues and blood from patients with moderate-to-severe AD and found that Th2 cytokines (IL5, IL13, CCL13, CCL18, and CCL26), as well as Th22 markers, IgE levels, and eosinophilic count significantly decreased with age in patients with AD. Moreover, expression of Th1-related (IFNG, IL12/23p40, STAT1, and CXCL9) and Th17-related (IL17A and IL20) markers increased with age in AD patients as well as in healthy controls [17]. To date, it remains to be determined whether these immunophenotypic differences represent specific molecular signatures related to the age and onset of AD or whether they are predominantly related to the evolution of the disease from acute to chronic. Current evidence suggests that these age-related changes in the AD molecular microenvironment are likely due to the complex interplay of multicytokine activation involved in long-standing chronic AD, which characterizes older patients [17]. The most accepted hypothesis is that the reduced Th2 activity with age in patients with AD could be due to counter-regulation by both Th1 and Th17, as well as by an increased regulatory tone [18]. This may ultimately promote disease attenuation or tolerance.

### 3.2. Extrinsic and Intrinsic AD

AD is classified into extrinsic or intrinsic according to high or normal IgE serum levels, respectively. The classic and more frequent extrinsic phenotype (80%) is characterized by high serum IgE levels, eosinophilia, personal and family atopic background, and greater rate of filaggrin (FLG) mutation [19], the latter being the strongest genetic risk factor for AD development [20].

Conversely, patients with the less frequent intrinsic phenotype of AD (20%) have normal IgE levels, have female predominance, show delayed disease onset, have increased metal contact hypersensitivity, and lack any other atopic backgrounds [21].

Specific immunological differences between extrinsic and intrinsic AD have been described and could partially explain the diversity across the two phenotypes. For example, one study comparing extrinsic vs. intrinsic AD immunological profiles found that Th2 marker increases were grossly similar in the skin of both groups of patients. However, increased Th1 signal (IFN-γ, CXCL9, CXCL10) and more pronounced Th17/Th22 activation (IL-17A, CCL20, Elafin, and IL-22) were significantly greater in patients with intrinsic AD, even suggesting immunological features of overlap with psoriasis [22].

### 3.3. Phenotypes According to Disease Stage

AD is clinically characterized by acute and chronic stages [23]. Acute lesions are usually erythematous, wet, and highly inflammatory, gradually becoming dry, lichenified, thick, and hyperpigmented in patients with chronic disease [24]. There is evidence for the different skin molecular milieu between acute and chronic AD lesions. In detail, one study evaluated gene expression profiles of AD lesions in different stages (acute vs. chronic) and found that acute disease was predominantly associated with significant increases in gene expression levels of major Th22- and Th2-cytokines, and smaller increases in IL-17 [25]. Conversely, significant increases in Th1-related products (i.e., IFN-γ, MX1, CXCL9-11) were detected only in chronic skin lesions [25]. Another study performed RNA-seq analyses to study the changes accompanying the transition from non-lesional to acute to chronic inflammation in AD. The gene expression data suggested that acute AD is primarily triggered through the action of IL-22, with smaller contributions from IL-17 and IFN-γ, accompanied by Th2 cytokines including IL-4/IL-13 and IL-31. In this study, the expression of Th2 and Th22 cytokines further increased in chronic lesions, and a higher activation of Th1 and Th17 responses was observed [26].

### 3.4. Phenotypes According to Ethnic Groups

Emerging evidence has highlighted that, depending on the patient’s racial background, AD seems to show different clinical, genetic, and immunopathogenic features [27]. 

In contrast to the well-described classical clinical picture in white AD patients, Asian individuals typically present lesions with more defined borders, sometimes closely resembling psoriasis plaques, as well as more scaling and lichenification [28,29]. Conversely, African AD patients present with a predominant extensor involvement [30] and sometimes with perifollicular accentuation and distinct papules on the extensor surfaces and on the trunk [31]. 

Differences in clinical manifestations are accompanied by heterogeneity in the immunological microenvironments across several ethnic groups [8]. Indeed, while the type 2 signal is constantly elevated in AD in all ethnic groups, an upregulation of other immune pathways can occur differently. In detail, the Asian endotype of AD is typically characterized by a strong Th17 signature, as well as by peculiar clinical and histological features resembling psoriasis [29,32]. Furthermore, a study on African American AD revealed a strong Th2/Th22-skewing, with both Th2 and Th22 markers correlating significantly with disease severity, and concomitantly, an attenuation of the Th1 and Th17 axes compared to European Americans [33].

## 4. Overview of the Market

With recent advances in research, a number of pharmaceutical agents have emerged as novel treatments for moderate-to-severe AD. 

In detail, currently approved drugs belong to two major classes, with different mechanisms of action: monoclonal antibodies targeting single or dual cytokines of the IL-4/13 pathway and broad-acting small molecule inhibitors (JAK inhibitors).

### 4.1. Approved Biologic Drugs Antagonizing the IL4/IL-13 Axis

Given the numerous effects of IL-4 and IL-13 in AD pathophysiology, including disruption of skin barrier, induction of bacterial binding and colonization, and recruitment of inflammatory cells, these cytokines and cytokine receptors have been attractive candidates for therapeutic targeting [34].

Dupilumab is a fully human monoclonal antibody blocking IL-4 and IL-13 by binding the shared IL-4 receptor α (IL-4Rα). It was the first AD-specific agent to be approved in both adults and adolescents with moderate-to-severe AD [35]. Dupilumab is now approved for AD in many countries, and real-world data support the efficacy reported in the phase III program [36,37]. As the drug was the first to be approved for the treatment of AD, dupilumab’s long-term efficacy and safety data have also been extensively studied. In detail, in a phase 3 open-label extension study, the drug demonstrated sustained efficacy up to 76 weeks of treatment with no new safety signals in comparison to clinical trials, with most common adverse events (AEs) being nasopharyngitis, conjunctivitis, and injection-site reactions [38]. Despite being a truly significant milestone in AD management, only 35% to 40% of patients on dupilumab achieve clear or almost clear skin (IGA score 0/1), thus endorsing the need for additional treatment options [39].

A few years after dupilumab entered the market, tralokinumab was approved, a fully human IgG4 monoclonal antibody that specifically binds to the type 2 cytokine IL-13 with high affinity, thereby inhibiting its interaction with IL-13 receptors and the IL-13Rα1/IL-4Rα receptor complex, and neutralizing the biological activity of IL-13 [40]. In two identically designed 52-week, multinational, randomized, double-blind, placebo-controlled trials (ECZTRA 1 and 2), achievement of an IGA score of 0 or 1 and EASI 75 at week 16 was significantly higher with tralokinumab vs. placebo. In detail, IGA0 or 1 was achieved by 15.8% with tralokinumab vs. 7.1% with placebo in ECZTRA 1 and by 22.2% with tralokinumab vs. 10.9% with placebo in ECZTRA 2 [41]. Long term efficacy and safety data of tralokinumab in AD were investigated in the ECZTEND open-label extension trial. The study showed that over 2 years, tralokinumab was well tolerated and able to maintain long-term control of AD signs and symptoms [42].

Very recently, lebrikizumab was approved for the treatment of moderate-to-severe AD [43]. The drug is a high-affinity IgG4 monoclonal antibody targeting IL-13, which prevents the formation of the IL-4Rα/IL-13Rα1 heterodimer receptor signaling complex. The drug approval followed the results from two phase III clinical trials (ADvocate 1 and ADvocate 2), in which patients were randomly assigned in a 2:1 ratio to receive either lebrikizumab at a dose of 250 mg (loading dose of 500 mg at baseline and week 2) or placebo, administered subcutaneously every 2 weeks. The primary outcome of IGA 0 or 1 was met in 43.1% of patients in the lebrikizumab group and in 12.7% of 141 patients in the placebo group in ADvocate 1, and in 33.2% of 281 patients in the lebrikizumab group and in 10.8% of 146 patients in the placebo group (*p* < 0.001 in both trials) [44]. As it is a newly introduced drug, studies on long-term efficacy and safety of lebrikizumab in AD are still ongoing. Both tralokinumab and lebrikizumab bind to soluble IL-13, although their mechanism of action slightly differs. Indeed, tralokinumab prevents access of the IL-13Rα1 and IL-13Rα2, while lebrikizumab interferes with IL-13 binding to IL-13Rα1, not IL-13Rα2, thus leaving the endogenous regulation of IL-13 levels through IL-13Rα2 intact [45]. The therapeutic implications of these different modes of actions are still to be fully elucidated.

### 4.2. Approved JAK Inhibitors

The JAK/STAT pathway is a paradigm of receptor-mediated signal transduction involved in several key biological processes, including cell proliferation, differentiation, apoptosis, and immune regulation [46]. This pathway has been investigated in many chronic inflammatory skin diseases, and its therapeutic inhibition has been therapeutically successful in some of these, such as AD, psoriasis, vitiligo, and alopecia areata [47,48]. Indeed, many of the key cytokines involved in AD pathogenesis, such as IL-4, IL-13, IL-31, and TSLP (Thymic Stromal Lymphopoietin), exert their functions through the activation of the JAK/STAT pathway [49].

Furthermore, the JAK/STAT pathway interacts with other critical pathways in skin immunology, such as TNFα signaling. In detail, STAT1 can be activated by TNFα, and the two pathways have been found to be closely connected in various diseases [50]. Specifically, TNFα was proven to induce spongiosis, augment TSLP secretion by keratinocytes, and alter early and terminal differentiation-protein expression in epidermal models [51]. Although it could be theorized that these effects might be mediated by the JAK/STAT signaling, TNFα inhibitors have not been proven successful for the treatment of AD, and eczematous reactions have even been described under TNFα inhibitors treatment [52]. Further studies are needed to elucidate the controversial role of TNFα in AD.

**Figure 1:**
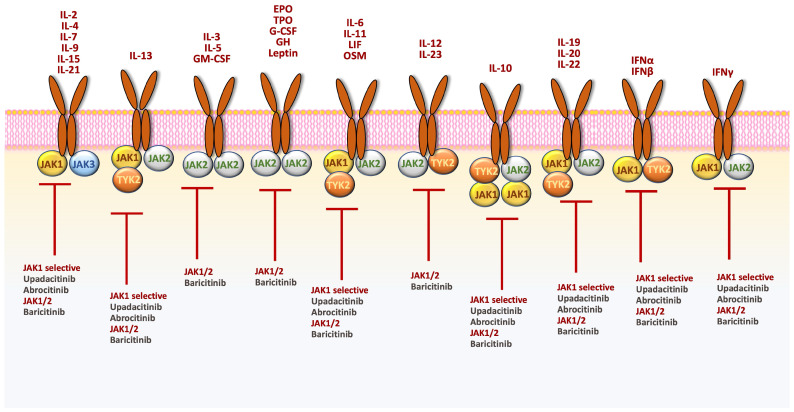
Schematic representation of the major cytokines that signal through the JAK/STAT pathways and the selectivity of JAK inhibitors approved for AD. AD, atopic dermatitis; INF, interferon; IL, interleukin; OSM, oncostatin M; LIF, leukemia inhibitory factor; GM-CSF, granulocyte-macrophage colony-stimulating factor; C-CSF, granulocyte colony-stimulating factor; EPO, erythropoietin; TPO, thrombopoietin; GH, growth hormone. EPO: erythropoietin; G-CSF: granulocite-colony stimulating factor; GH: growth hormone; GM-CSF: granulocite macrophage-colony stimulating factor; IFN: interferon; LIF: leukemia inhibitory factor; OSM: oncostatin M; TPO: thrombopoietin.

To date, three JAK inhibitors (JAKi), with different selectivity toward JAK, have been approved for the treatment of AD in Europe: upadacitinib, abrocitinib, and baricitinib [53] (Figure 1).

Upadacitinib (UPA) is an orally administered selective JAK1 inhibitor approved for the treatment of moderate-to-severe AD [54]. The drug is a reversible ATP competitive inhibitor with a much higher selectivity for JAK1 than for JAK2, JAK3, or TYK2 (IC50 0.045, 0.109, 2.1, and 4.7 μmol/L) [55]. In MeasureUp 1 and 2 phase III trials, patients were randomly assigned (1:1:1) to receive UPA 15 mg, UPA 30 mg, or placebo once daily for 16 weeks; a significantly higher proportion of patients achieved the primary endpoints of IGA0/1 at week 16 in the UPA groups in comparison to placebo [MeasureUp1: UPA 15 mg (48%); UPA 30 mg (62%) vs. placebo (8%)] [MeasureUp2: UPA 15 mg (39%); UPA 30 mg (52%) vs. placebo (5%)]. The analysis of follow-up data from Measure Up 1 and 2 showed that longer-term treatment with UPA had a favorable benefit–risk profile with sustained efficacy responses through 52 weeks [56]. Furthermore, an integrated analysis of safety data from phase III clinical trials over a span of up to 5 years demonstrated that the incidence of adverse events (AEs) remained low throughout treatment with UPA, supporting a favorable benefit–risk profile [57].

Abrocitinib (ABRO) is an orally administered, selective JAK1 inhibitor which has recently received EMA and FDA approvals for the treatment of adults with moderate-to-severe AD [58]. The drug demonstrated efficacy in AD in two phase III clinical trials (JADE MONO-1, JADE MONO-2), in which both ABRO 200 mg and 100 mg met the co-primary endpoints at week 12 [JADE MONO-1: ABRO 100 mg (24%); ABRO 200 mg (44%) vs. placebo (8%)], [JADE MONO-2: ABRO 100 mg (28.4%); ABRO 200 mg (38.1%) vs. placebo (9.1%)] [59,60]. To date, 48-week efficacy and safety data of ABRO in AD have demonstrated a sustained clinical response along with a manageable safety profile [61]. Furthermore, an interim analysis of the JADE-EXTEND trial confirmed a clinically meaningful improvement in signs and symptoms of AD in patients treated with ABRO [62,63].

Baricitinib (BARI) is an orally administered small molecule, a selective inhibitor of JAK1 and JAK2 tyrosine kinases. Recently, the drug was approved in Europe and Japan in adult patients with moderate-to-severe AD at both 4 and 2 mg oral daily dosage [64,65]. The efficacy and safety of the drug were investigated in a wide phase III clinical trials program. In detail, in BREEZE-AD1 and BREEZE-AD2, BARI monotherapy (4 mg, 2 mg, or 1 mg) was compared to placebo in adults age ≥ 18 years with moderate-to severe AD. In both studies, the primary endpoint of validated IGA 0/1 was met by a significantly higher proportion of patients in the 2-mg and 4-mg groups, compared to placebo [BREEZE-AD1: BARI 4 mg (16.8%); BARI 2 mg (11.4%) vs. placebo (4.8%)], [BREEZE-AD2: BARI 4 mg (13.8%); BARI 2 mg (10.6%) vs. placebo (4.5%)] [66]. The long-term efficacy of baricitinib combined with topical corticosteroids (TCS) showed a maintained clinically meaningful sustained efficacy of both BARI dosages over 68 weeks of continuous treatment [67]. Furthermore, an integrated analysis of eight BARI clinical trials in AD reported a tolerable safety profile with no new safety signals in comparison to previous reports up to 3.9 years of treatment [68]. 

## 5. Factors Driving Therapeutic Choice between Monoclonal Antibodies and JAK Inhibitors

With the increasing number of options for the treatment of moderate-to-severe AD, there is a need for a precise guidance on a practical approach to selecting a systemic agent for specific patient populations.

**Figure 2:**
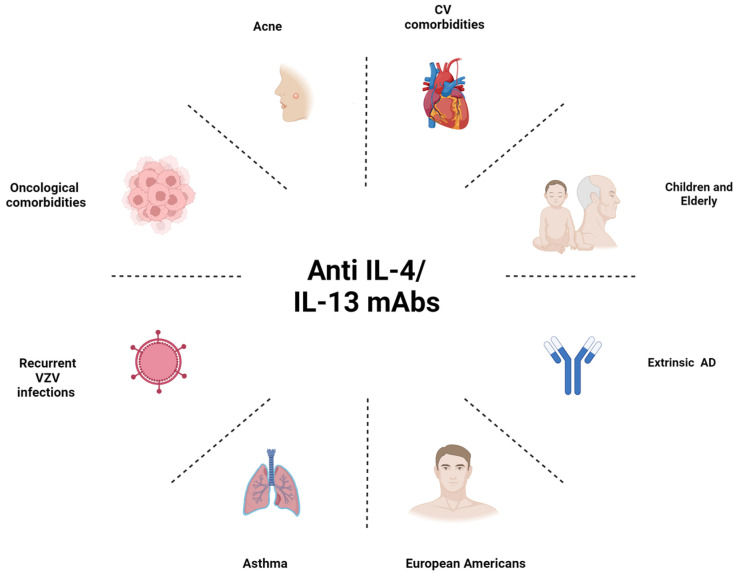
Schematic representation of the special AD populations and main factors that could orient the clinician’s choice towards a monoclonal antibody targeting the IL-4/13 axis.

**Figure 3:**
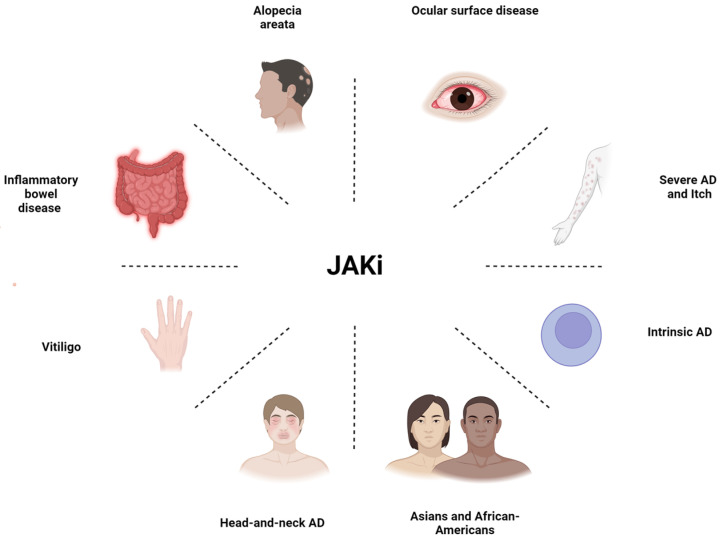
Schematic representation of the special AD populations and main factors that could orient the clinician’s choice towards a JAK inhibitor.

Indeed, the choice of the most appropriate systemic agent to be used for the management of moderate to severe AD is not straightforward, and several important factors have to be considered, such as the heterogeneity of AD and its classification in phenotypes, patients’ comorbidities, age, and personal preferences (Figure 2 and Figure 3).

### 5.1. AD Clinical Phenotypes and Safety Concerns

As mentioned above, an increasing number of AD clinical phenotypes have been described. Recently, in a prospective practice-based study on 592 AD patients, Chovatiya et al. proposed a classification of AD, combining itch and lesional severity into four phenotypes: mild–moderate itch and lesions (MI-ML), mild–moderate itch and severe lesions (MI-SL), severe itch and mild–moderate lesions (SI-ML), and severe itch and lesions (SI-SL) [69]. A post hoc analysis of pooled data from clinical trials was recently conducted in order to evaluate ABRO efficacy in patients who had an itch-dominant phenotype of AD, defined as a baseline Peak Pruritus Numerical Rating Scale (PP-NRS) score of 7–10. The results showed that most of these patients experienced itch improvement over time with ABRO monotherapy or with concomitant topical therapy [70]. Furthermore, selective JAK1 inhibitors, UPA and ABRO, were directly compared to dupilumab in two phase III head-to-head trials, namely, Heads Up and JADE-DARE, respectively [71,72]. In detail, in the Heads Up trial, the most significant differences between UPA 30 mg and dupilumab were found in the rapidity of onset and the ability to better achieve high levels of skin clearance (i.e., EASI90 and EASI100) and itch improvement, with significantly higher rates of clinically meaningful reduction in itch reported as early as week 1 [71]. Similarly, in the JADE-DARE trial, ABRO 200 mg provided higher amounts of early itch reduction than dupilumab (PP-NRS4 response at week 2) and a faster onset of high-level improvement of disease signs (EASI90 response at week 4) [72].

From all this evidence, we could infer that JAKi might be preferred in AD patients with itch-dominant phenotypes of AD, where a rapid therapeutic outcome is required.

When it comes to the choice between JAKi, little data have been available so far. To date, there are no head-to-head clinical trials between JAKi in AD. Evidence from network meta-analyses comparing different systemic treatments for AD showed that UPA 30 mg daily was the most efficacious targeted therapy, followed by ABRO 200 mg daily and UPA15 mg daily, after 12 or 16 weeks of therapy [73,74]. However, these results must be taken with caution considering all the limitations of a network meta-analysis, which is not a substitute for a head-to-head comparison.

On the other hand, data from the use of oral JAKi in immune-mediated disorders, such as rheumatoid arthritis (RA), have raised safety concerns, suggesting potentially increased risks of infection (especially Herpes Zoster), venous thromboembolism (VTE), and malignancy [75]. It is worth noting that these safety issues stemmed mainly from the use of tofacitinib (a pan JAKi) in a population already enriched for cardiovascular diseases (RA patients) and were not confirmed in clinical trials of selective JAK1 inhibitors in AD [76,77]. Nevertheless, the EMA recommends that JAKi should only be used if no suitable treatment alternatives are available in patients 65 years of age or older, in patients with history of atherosclerotic cardiovascular (CV) disease or other CV risk factors, and in patients with malignancy risk factors [77,78]. In such cases, mAbs should be preferred. In this context, a real-world study on 155 adult AD patients, including those with significant comorbidities such as malignancies, reported dupilumab to be an effective and safe option [79]. Evidence on the safety of dupilumab in elderly AD patients has also been reported [80]. Furthermore, a post hoc analysis for adults 65 years or older was conducted from tralokinumab phase 3 trials (ECZTRA 1, 2 and ECZTRA 3), suggesting that the drug is well tolerated and efficacious in elderly AD patients [81]. Finally, a network meta-analysis compared the incidence and risk of herpes zoster among patients with moderate-to-severe AD treated with advanced systemic therapies and found that JAK1 inhibitors are associated with a significantly higher incidence compared to dupilumab and placebo [82]. However, further studies are still needed to better delineate and compare the long-term safety of mAbs and JAKi in “fragile” AD populations.

### 5.2. Body Areas

Of note, the “head-and-neck” phenotype of AD has proven to be refractory to dupilumab therapy in real-world studies [83,84]. For example, a study conducted on 347 AD patients treated with dupilumab for 104 weeks reported that AD in the head-and-neck area remained present in most patients at high levels; the proportion with head-and-neck AD at baseline was 76% and 68% at week 104 [83]. Interestingly, cases of “head-and-neck” AD refractory to dupilumab have been shown to benefit from switching to a JAKi [85,86].

Moreover, the use of dupilumab has even been associated with the induction of a paradoxical facial erythema also referred to as DAHND (dupilumab-associated head and neck dermatitis) [87,88,89], which has been reported to resolve after transition to JAKi [90,91]. Therefore, it might be suggested that in cases of predominant localization of AD to the head-and-neck area, the therapeutic choice might reside with JAKi instead of mAbs [92].

### 5.3. Comorbidities

Notably, the existence and the types of comorbidities may be important elements to consider in the choice of the most appropriate therapeutic strategy. If, as mentioned above, oncological or cardiovascular comorbidities may guide the clinician toward the choice of an mAb, the co-occurrence of AD with other immune-mediated dermatological conditions, such as psoriasis, alopecia areata (AA), vitiligo, or even hidradenitis suppurativa, may be an argument in favor of choosing a JAKi. In fact, the coexistence of immune-mediated skin disorders is not uncommon [93,94], and JAKi has demonstrated clinical efficacy on many of these diseases as well [48,95,96,97,98]. In particular, differences in labeling indications might guide the choice of the most appropriate drug in clinical practice. For instance, beside AD, UPA is approved for the treatment of RA, axial spondyloarthritis, Crohn’s disease (CD), and ulcerative colitis (UC); it might, therefore, be considered as a preferred option in AD patients with these conditions [54]. 

Similarly, BARI is approved for the treatment of AA, RA, and juvenile idiopathic arthritis and could, therefore, be the best choice in such cases [99]. With regard to concomitant AD and RA, as both UPA and BARI are approved, the choice between the two should be weighted according to individual patient-related factors.

On the other hand, the frequent association of AD with allergic comorbidities, such as asthma, could guide the clinician towards the choice of an mAb. In particular, dupilumab has received EMA approval for asthma in patients over 6 years of age, chronic rhinosinusitis with nasal polyposis, prurigo nodularis, and eosinophilic esophagitis, and it could be envisaged as a preferred treatment option in patients with AD plus one of these conditions [35]. Conversely, tralokinumab has not demonstrated efficacy in treating asthma, likely due to the overlapping function between IL-4 and IL-13 [100,101]. 

Interestingly, biologics for AD that involve Th2 blockade via inhibition of IL-4 and/or IL-13 were associated with an increased incidence of ocular AEs, which led to the introduction of the new term: medication-induced ocular surface disease (mOSD) [41,102,103]. Notably, AD itself is associated with an increased risk of developing OSD, including conjunctivitis, keratitis, and keratoconus [104]. Head-to-head studies on ABRO [105] and UPA [71] versus dupilumab demonstrated lower rates of ocular AEs in the JAKi-treated patients. Therefore, for patients with a history of severe OSD, dermatologists could consider preferentially prescribing a JAKi, instead of an mAb to proactively avoid the possibility of severe OSD [92].

Finally, the use of JAK inhibitors has been associated with a high risk of developing acne or acneiform reactions, as well as worsening pre-existing acne [106]. Head-to-head studies of JAKi versus dupilumab demonstrated lower rates of acne in the dupilumab-treated patients [71,105]. Accordingly, it might be suggested that in patients with concomitant AD and acne, the choice of systemic treatment might better fall on an mAb rather than a JAKi.

### 5.4. Patients’ Stratification in Endotypes

The heterogeneity of AD extends beyond the clinical features of the disease to its molecular profiles [4], and clinical phenotypes do not necessarily relate to the underlying disease’s mechanism or molecular markers. Therefore, the identification of biomarkers defining distinct molecular endotypes could result in better characterization and stratification of AD patients as well as provide guidance toward the most appropriate therapeutic choice.

Several attempts have been made to identify the molecular signatures of disease subtypes and the driver cytokines/cell-types thereof. Various ways of endotyping patients with AD have been described, the most common being based on the identification of serum and/or tissue biomarkers [107]. For example, Thijs et al. were able to classify adult patients with AD into four distinct patient clusters based on serum biomarker profiles [108]. In another study, Bakker et al. performed proteomic analysis on AD sera and again identified four serum biomarker–based clusters [109], three of which were comparable to those identified in the previous study [110]. In detail, these were cluster B, identified as a “Th1/Th2/Th17-dominant” cluster; cluster C (18.5%), a “Th2/Th22/PARC (pulmonary and activation-regulated chemokine)-dominant” cluster; and cluster D (29.5%), a “Th2/eosinophil-inferior” cluster [109].

In a recent study, Sekita et al. performed an integrated analysis of RNA-seq data from skin tissue and peripheral blood mononuclear cell (PBMC) samples from 115 AD patients and 14 healthy controls in order to identify phenotype-endotype associations [111]. The authors identified a correlation between two main qualitatively differential skin manifestations of AD, erythema and papulation, and two different immunological signatures (endotypes). Furthermore, they described three patient clusters based on blood-derived signatures closely linked to a different disease course and medical history, and, in detail, cluster 1 showed severe and stable symptoms, cluster 2 showed severe and unstable symptoms, and cluster 3 showed mild symptoms [111].

In addition to defining the disease endotype, tissue or blood biomarkers may be useful in predicting whether a patient population may benefit from a certain therapy (predictive biomarkers). In this context, Glickman et al. found that baseline gene expression levels of the Th17-related cytokine CXCL2 in the skin from AD patients showed strong predictive responses for dupilumab treatment, thus proposing this molecule as a predictive biomarker [112]. 

Overall, these studies confirm how endotypes can provide important information on individualized treatment options as well as represent a step forward towards a more precise stratification of AD patients and to the identification of novel phenotype–endotype correlations. Unfortunately, due to the lack of validated biomarkers in clinical practice, patients cannot be assigned to a specific treatment yet. However, based on the current insights, some assumptions can be made.

For instance, anti-IL-4/IL-13 mAbs could theoretically exert a beneficial effect on Th2-dominant AD subtypes such as extrinsic AD, European American AD, or AD in children. Conversely, patients with intrinsic AD, Asian and African-American AD patients in whom multi-axis activation with Th22 and Th1 contribution is demonstrated, even though it is to varying degrees, might benefit from a broad-acting agent, such a JAKi.

However, further experimental and observational studies are needed to confirm whether these assumptions can actually be reflected in clinical practice.

## 6. Conclusions

With an increase in therapeutic options for the treatment of moderate-to-severe AD, understanding heterogeneity in disease phenotypes and endotypes as well as patient stratification are the two urgent tasks for the development of personalized medicine in AD. The introduction of mAbs targeting IL4/13 and JAKi has not only represented a breakthrough in the treatment of moderate-to-severe AD, but it has also provided important insights into AD pathogenesis. Given the high heterogeneity of AD in terms of clinical course and response to treatment, it could be assumed that certain disease subtypes may respond better to one approach than to another, thus providing a guide for therapeutic choice in clinical practice. Moreover, the presence of certain AD comorbidities may also be a driver in the choice of one drug class over another.

From the current knowledge we have on the mechanism of action of different drugs, clinical trial data, and real-world studies, it could be inferred that an itch-dominant phenotype of AD, the presence of “head-and-neck” AD, OSD, or immune-mediated comorbidities, could be oriented toward a JAKi. On the other hand, in fragile populations (i.e., elderly), the presence of oncologic, cardiovascular or allergic comorbidities, recurrent herpes zoster infections, or acne vulgaris could orient the physician toward an anti IL4/13 monoclonal antibody. However, a step forward would be to base the choice between the two main therapeutic strategies, IL-4/IL-13 or JAK inhibition, on precise molecular profiling of patients, namely, endotypes. Indeed, as we are moving toward an era of more targeted therapies and we have acknowledged the extreme heterogeneity of AD, the application of molecular biomarkers should be pursued, as it will result in a better characterization and stratification of patients. This systematic approach will allow us to better compare current and new treatments as well as to stratify patients on the basis of their immunological drivers (endotypes), thus paving the way for a novel, tailored, endotype-driven therapeutic choice. Interestingly, in recent years, novel mechanisms of action that indirectly interfere with multiple T cell populations simultaneously (e.g., Th1, Th2, Th17, Th22) are being investigated in AD. Specifically, monoclonal antibodies targeting the transmembrane glycoprotein OX40 receptor (OX40), such as rocatinlimab, and its ligand OX40L, such as amlitelimab, have shown encouraging results in phase II studies. These treatments are currently being investigated in phase III clinical trials for moderate-to-severe AD [113,114].

## Data Availability

Not applicable.
